# The histone modification H3K4me3 is altered at the *ANK1* locus in Alzheimer's disease brain

**DOI:** 10.2144/fsoa-2020-0161

**Published:** 2021-02-09

**Authors:** Adam R Smith, Rebecca G Smith, Ruby Macdonald, Sarah J Marzi, Joe Burrage, Claire Troakes, Safa Al-Sarraj, Jonathan Mill, Katie Lunnon

**Affiliations:** 1 University of Exeter Medical School, University of Exeter, Exeter EX2 5DW, UK; 2 The Blizard Institute, Queen Mary University of London, London E1 2AT, UK; 3 Institute of Psychiatry, Psychology & Neuroscience, King's College London, London SE5 8AF, UK

**Keywords:** Alzheimer's disease, *ANK1*, brain, chromatin modifications, DNA methylation, Epigenetics, histone modifications, H3K4me3, H3K27me3

## Abstract

Several epigenome-wide association studies of DNA methylation have highlighted altered DNA methylation in the *ANK1* gene in Alzheimer's disease (AD) brain samples. However, no study has specifically examined *ANK1* histone modifications in the disease. We use chromatin immunoprecipitation-qPCR to quantify tri-methylation at histone 3 lysine 4 (H3K4me3) and 27 (H3K27me3) in the *ANK1* gene in entorhinal cortex from donors with high (n = 59) or low (n = 29) Alzheimer's disease pathology. We demonstrate decreased levels of H3K4me3, a marker of active gene transcription, with no change in H3K27me3, a marker of inactive genes. H3K4me3 is negatively correlated with DNA methylation in specific regions of the *ANK1* gene. Our study suggests that the *ANK1* gene shows altered epigenetic marks indicative of reduced gene activation in Alzheimer's disease.

## Lay abstract

Alzheimer's disease affects millions of people worldwide and numerous genetic association studies have been undertaken in the disease in recent years. The expression of genes can be altered by epigenetic processes, which include modifications to the DNA or histone proteins. Several studies have now reported increased DNA methylation in the *ANK1* gene in Alzheimer's disease brain samples. However, to date no studies have explored histone modifications in this gene in the disease. Here, we show decreased levels of the H3K4me3 modification in regions of the *ANK1* gene in the entorhinal cortex of Alzheimer's disease brain samples, which is a marker of active gene expression. This study further supports a role for epigenetic modifications in the *ANK1* gene in Alzheimer's disease pathology.

## Graphical abstract

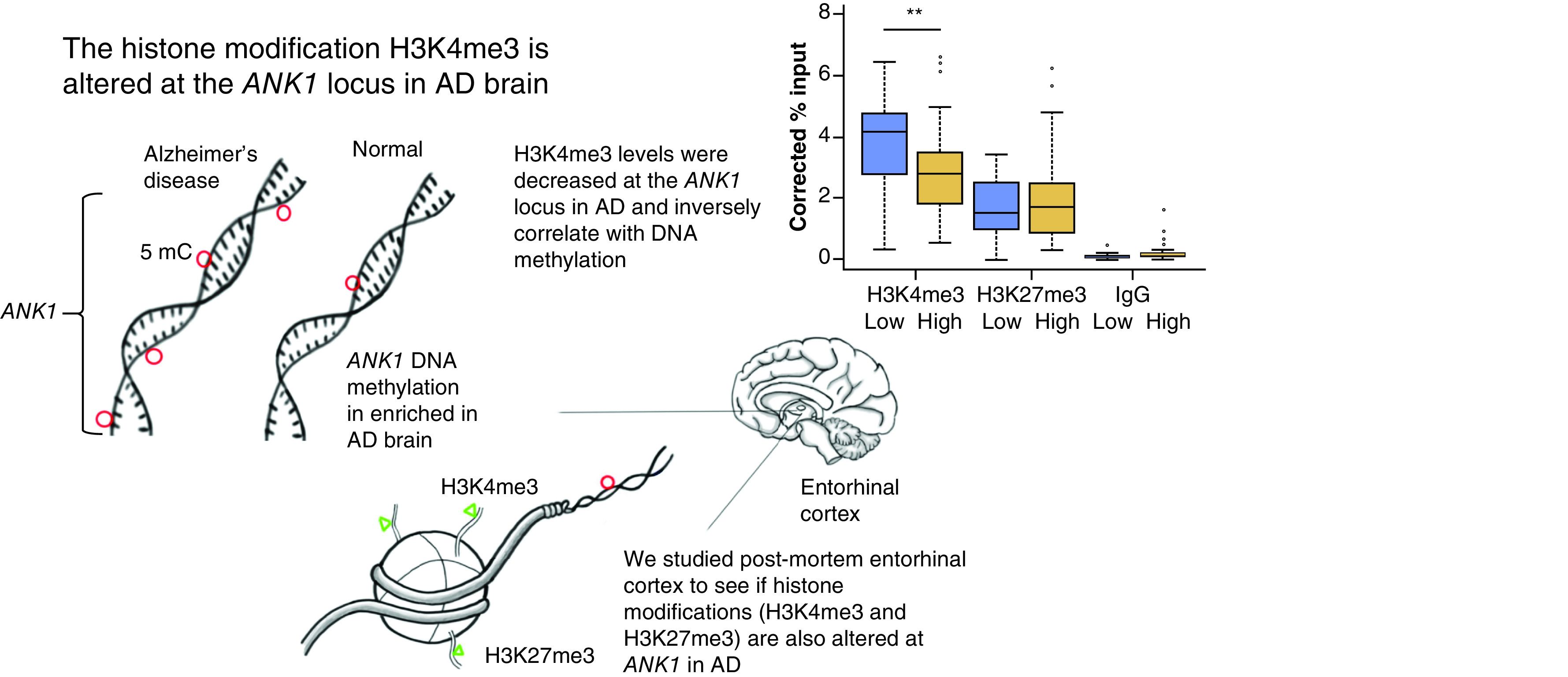

Alzheimer's disease (AD) is a chronic neurodegenerative disease affecting ∼30 million people worldwide, with this figure predicted to continue rising [1]. The disease is characterized by the accumulation of amyloid-β (Aβ) plaques and neurofibrillary Tau tangles within the brain. Clinically, AD manifests as a reduction in the ability to retain new information, difficulty in planning and solving problems, confusion, mood and personality changes. By the time an individual becomes symptomatic, there is already considerable neuropathology, which can appear years before the clinical diagnosis. An improved understanding of the underlying mechanisms driving disease onset and progression is required to enable the design of new, more effective medications.

AD has been suggested to have an epigenetic contribution to disease etiology [2–4]. In recent years a number of epigenome-wide association studies (EWAS) have highlighted altered DNA methylation at a number of loci in AD brain samples [5–9], with a recent meta-analysis of ∼1400 individuals highlighting consistent alterations in several genes [10]. Hypermethylation of a region spanning ~100 bp of the *ANK1* gene is one of the most reproducibly nominated loci in AD cortex [2,10]. Furthermore, it has been highlighted that DNA methylation changes in the *ANK1* gene in the entorhinal cortex (EC) are observed in AD, Huntington's disease and to some extent in Parkinson's disease donors, but not in individuals with pure vascular dementia or dementia with Lewy bodies in the absence of co-existing AD pathology [11].

Histone modifications represent another epigenetic mechanism, which mediates the reversible short-term regulation of hetero/euchromatin [12]. The level of gene transcription can be modulated by the presence of histone modifications including methylation, phosphorylation, acetylation, ubiquitination, sumoylation, citrullination and adenosine diphosphate-ribosylation [13]. Two of the most studied histone modifications are the tri-methylation of lysine at positions 4 and 27 of histone 3 (H3K4me3 and H3K27me3), which are markers of gene expression and repression, respectively. H3K4me3 is consistently present at transcription start sites (TSS) of actively transcribed genes in eukaryotes, with high levels of the modification reflecting the amount of transcription [14]. H3K4me3 is also localized at the 5′ end of active genes near the TSS and has been shown to be associated with the initiated form of RNA polymerase II [15]. Unlike H3K4me3, H3K27me3 is associated with inactive gene promotors, causing a dramatic and predictable lack of gene expression [15–17] and is involved in the maintenance of the inactive X-chromosome in females and in genomic imprinting [18]. H3K4me3 and H3K27me3 can occur on the same histone tail: these so-called ‘bivalent modifying patterns’ mean that viewing modifications as ‘activating’ or ‘silencing’ is overly simplistic. These bivalent patterns in embryonic stem cells (ESC) are predicted to cause highly dynamic levels of gene expression [19].

Histone modifications have been associated with neurological diseases, such as AD. Cleavage of the amyloid precursor protein in AD leads to the production of amyloid precursor protein intracellular domain (AICD) which, coupled with Fe65, activates TIP60, a histone acetyltransferase. This acetyltransferase leads to increased acetylation of H3K14 and H4K5 [20]. In addition, work in mice has revealed that neuron-specific overexpression of histone deacetylase 2 (HDAC2) decreases dendritic spine density, synapse number, synaptic plasticity and memory function. However, HDAC2 deficiency was shown to cause an increase in synaptic number and memory facilitation [21]. Although the majority of EWAS in AD have focussed on DNA methylation to date, two recent EWAS have profiled histone acetylation in AD post-mortem brain tissue using Chromatin Immunoprecipitation Sequencing (ChIP-seq) [22,23]. Marzi *et al.* studied acetylation of H3K27 (H3K27ac), a marker of active enhancers and gene expression, identifying widespread AD-associated variation [23]. Differentially acetylated peaks were observed in genomic regions associated with both tau and amyloid neuropathology and genes harbouring variants associated with AD neuropathology, for example, *APP*, *PSEN1*, *PSEN2* and *MAPT*. Similarly, Klein *et al.* profiled acetylation of H3K9 (H3K9ac) in a large cohort of 669 individuals, identifying a number of altered regions with respect to Aβ or tau protein burden [22]. In particular, with respect to tau pathology they observed large genomic alterations in H3K9ac, suggesting genome-wide chromatin reorganization. These studies further support a role for alterations in histone modifications in neurological diseases, such as AD.

To date, only one study has specifically examined histone modifications in *ANK1*, by interrogating the ENCODE ChIP-seq dataset [19]. This study focused on the proposed erythrocytic specific promotor of *ANK1* and the level of enhancer-associated histone modifications in this region (H3K4me1 and H3K27ac) [24]. However, this approach was limited to a human blood cell line (K562 cells) and therefore this study has limited utility for aiding the understanding of *ANK1* histone modifications in brain tissue. At present, no study has examined alterations in H3K4me3 and H3K27me3 at the *ANK1* locus in AD brain tissue. To assess the histone modification profile of the *ANK1* gene in AD and to see if there was any correlation with the previously reported DNA hypermethylation at this locus, this study utilized ChIP quantitative PCR (ChIP-qPCR) to quantify H3K4me3 and H3K27me3 modifications in the *ANK1* gene in post-mortem brain samples from donors with varying degrees of AD pathology.

## Materials & methods

### Subjects & samples

This study used EC tissue from post-mortem brain samples collected from 88 individuals archived in the MRC London Neurodegenerative Diseases Brain Bank. We had matched genome-wide DNA methylation and hydroxymethylation data generated for 63 of these samples using the Illumina Infinium Human Methylation 450K BeadChip Array (450K array) on bisulfite and oxidative-bisulfite treated DNA samples, which is available from the gene expression omnibus under accession number GSE105109 [5]. In addition, a subset of 47 samples had matched ChIP-seq data for the modification H3K27ac [23]. Donors used in the current study had varying degrees of AD pathology, ranging from Braak Stage 0 to Braak Stage VI, and were all over the age of 65 years. All samples were dissected by trained specialists, snap-frozen and stored at -80°C. Further demographic information about all samples is provided in Supplementary Table 1.

### ChIP for H3K4me3 & H3K27me3

H3K4me3 and H3K27me3 profiling was performed using the TrueMicroChip Kit from Diagenode (cat no.: C01010130). Briefly, frozen post-mortem brain tissue samples were cut into 10–15 mg sections. Subsequently, DNA and associated proteins were cross-linked using formaldehyde and cells were then resuspended in Higgs balanced salt solution (HBSS). Cells were incubated in lysis buffer and sonicated for 35 min to shear the chromatin (high: 30 s on 30 s off). After fragmentation, each chromatin sample was analysed via gel electrophoresis to check the size of the chromatin fragments were between 100 and 600 bp. Following this, automated magnetic immunoprecipitation was used for each chromatin sample, using three separate antibodies (100 μl of chromatin per antibody): H3K4me3 (Diagenode, cat no.: C15410003), H3K27me3 (Diagenode, cat no.: C15410069) and an IgG negative control (Diagenode, cat no.: C15400001). Finally, DNA was de-crosslinked from the protein and was purified using spin columns (Diagenode, cat no.: C03040001) and eluted in 80 μl elution buffer, leaving pure DNA for downstream qPCR.

### qPCR

qPCR was used to detect specific DNA target sequences in *ANK1*. A total of 2 μl ChIP-treated DNA was combined with 2 μl EvaGreen master mix (SolisBiodyne, cat no.: 08-24-00008), 2–3 μM forward and reverse primers and 5 μl of H_2_O per sample replicate in 384 well plates. Seven, one in four serial dilutions and a no template control (NTC) were included in triplicate per plate. The resulting plate was run on a QuantStudio (Thermo Fisher, US) qPCR machine with the following settings: 95°C for 10 min, 45 cycles of: 95°C for 30 s, 60°C for 30 s, 72°C for 30 s, followed by a dissociation stage.

### *ANK1* ChIP-qPCR primer design

ChIP-qPCR primers were designed using Primer3Plus software [25]. Primers were designed to target the core region based on Illumina 450K array probe location, previously shown to be differentially methylated in AD (chr8: 41519302 to 41519420) [7], across the remainder of the *ANK1* gene focussing on regions characterized by high H3K4me3 and H3K27me3 abundance and to other regulatory regions of the *ANK1* gene (Supplementary Figure 1). In total six primer sets met our inclusion threshold of producing a single gene product and with 85–115% efficiency. Therefore, six *ANK1* primer sets and one control primer set (MyoD1) [26] were used in this study (Supplementary Table 2).

### Data analysis

All samples were run in triplicate for each assay and averaged to collect a mean Ct score for each modification. A range of 0.5 Ct maximum was allowed between triplicate readings; Ct scores that fell outside this range were excluded and then an average of the two remaining scores was used. A control assay (MyoD1) with a stable histone modification profile was also run for all samples (Supplementary Figure 2). In addition, a negative control IgG ChIP reaction was run for all samples in all assays, as per the manufacturer's recommendations. Standard curves of a one in four dilution series were run on each plate and samples were normalized according to primer efficiency.

All computations and statistical analyses were performed using R 3.4.3 [27]. A linear regression model was performed to compare samples with a lower neuropathological burden (Braak 0–III, n = 29) with samples with a higher neuropathological burden (Braak IV–VI, n = 59) in each region covered by a primer set, controlling for the effects of age and sex. We then used a paired t-test to assess the difference in the mean levels of histone modifications averaged across all six primer regions between low (Braak 0–III) and high (Braak IV–VI) neuropathological burden. A Bonferroni significance threshold of p < 8.3 × 10^-3^ was used to account for multiple testing (six tests). A Pearson's correlation was used to compare histone modification levels with 5-methylcytosine (5 mC) and 5-hyroxymethylcytosine (5 hmC) levels in *ANK1* in the subset of 63 samples who had Illumina 450K array data already available [5]. A Bonferroni significance threshold of p < 4.67 × 10^-4^ was used to account for multiple testing (107 tests).

To compare our data with matched H3K27ac ChIP-seq data that had been previously generated in these samples we used the aligned reads from Marzi *et al.* [23] and generated read counts per primer region with htseq. These were then normalized as counts per million (CPM) to the total library sizes and we then subsequently performed a correlation analysis of H3K27ac CPM with either H3K4me3 or H3K27me3 levels for each of the six primer regions whilst controlling for age and sex to investigate whether there was an association between H3K27ac and H3K4me3 or H3K27me3 across all samples. Next, we included pathology group in the model as an interaction term to explore whether there was an association of H3K27ac and H3K4me3 or H3K27me3 specifically in only the low pathology or high pathology groups. A Bonferroni significance threshold of p < 8.3 × 10^-3^ was used to account for multiple testing (six tests for each of the modifications).

## Results

### *ANK1* has histone modification peaks at known regulatory elements

We started by examining the publicly available data from ENCODE for the *ANK1* region in frontal cortex for both H3K4me3 and H3K27me3 modification levels. This highlighted six key ‘peaks’ in H3K4me3 levels across the gene, with no obvious ‘peaks’ in H3K27me3 levels (Supplementary Figure 1). Our primer sets overlap four of these ‘peaks’ in H3K4me3 levels. In addition, many H3K4me3 ‘peaks’ overlap with known regulatory elements, including enhancers and promotors as well as TSS for several RNA transcripts. We also note that the previously identified *ANK1* differentially methylated region in AD [7] sits within the largest regulatory element of the *ANK1* locus. It is likely that histone modification differences at these key locations will have functional implications on expression, not only of *ANK1* but also of other RNA transcripts that have overlapping regulatory elements.

### *ANK1* has decreased levels of H3K4me3 in individuals with high neuropathology

**Figure 1.**
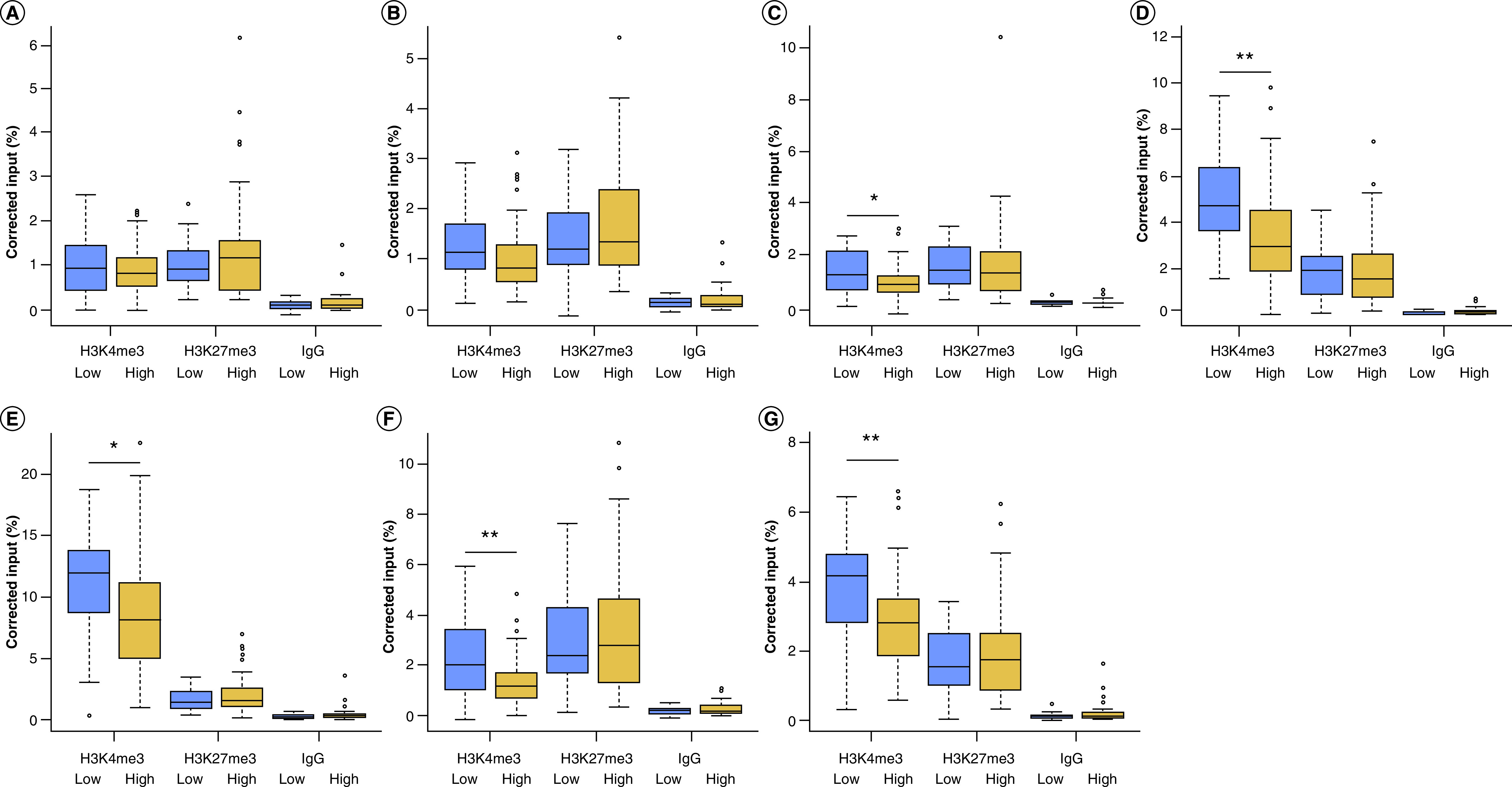
*ANK1* has decreased levels of H3K4me3 in individuals with high AD neuropathology. Boxplots of levels of H3K4me3, H3K27me3 and IgG control (shown as corrected percentage relative to input, after correction for age and sex). **(A)** We observed no change in H3K4me3 at Chr8:41516703-41516777 (primer set 1) or **(B)** Chr8:41519216-41519359 (primer set 2). We observed a nominally significant decrease in H3K4me3 in individuals with high levels of pathology compared with low levels of pathology at **(C)** Chr8: 41519342-41519460 (primer set 3, H3K4me3 difference p = 0.033) and **(E)** chr8: 41686181-41686328 (primer set 5, H3K4me3 difference p = 0.023), with a Bonferroni significant decrease in H3K4me3 in individuals with high pathology compared with low pathology at **(D)** Chr8: 41625416-41625491 (primer set 4, H3K4me3 difference p = 0.004) and **(F)** chr8: 41754877-41755012 (primer set 6, H3K4me3 difference p = 0.002), with the regions covered. We found no significant different for H3K27me or the IgG control for any of the genomic regions tested. **(G)** When we averaged histone modification levels across the six regions of *ANK1* that we examined, we observed a Bonferroni significant reduction in H3K27me3 in individuals with a high neuropathology burden, compared with those with a low burden (p = 0.0043), with no change in H3K27me3 or IgG control. *p < 0.05; **p < 0.01; ***p < 0.005.

We assessed levels of H3K4me3 and H3K27me3 across the *ANK1* gene in the EC using the six qPCR assays. Of the six regions assayed, two showed a nominally significant decrease in the level of H3K4me3 in individuals with high compared with low AD neuropathological burden, at the genomic coordinates chr8:41519342-41519460 (primer Set 3: Figure 1C, fold change [FC] = -0.30, p = 0.033) and chr8:41686181-41686328 (primer Set 5: Figure 1E, FC = -0.22, p = 0.023), with two regions showing a Bonferroni significant decrease (p < 8.3 × 10^-3^), at the genomic coordinates chr8:41625416-41625491 (primer Set 4: Figure 1D, FC = -0.31, p = 0.004) and chr8:41754877-41755012 (primer Set 6: Figure 1F, FC = -0.39, p = 0.002); with no significant difference in the other two regions tested (Figure 1A & B). We observed no significant difference in either H3K27me3 or the IgG control between individuals with low and high levels of pathology, in any of the six regions examined (Supplementary Table 3; Figure 1). Averaging histone modification levels across all six regions tested demonstrated a Bonferroni significant decrease in H3K4me3 in cases with higher AD neuropathology compared with those with lower neuropathological burden (FC = -0.25, p = 0.004), with no change in H3K27me3 (FC = 0.10, p = 0.52) or the IgG control (FC = 0.61, p = 0.21) (Figure 1G).

### H3K4me3 & H3K27me3 levels are correlated in post-mortem brain samples

H3K4me3 and H3K27me3 histone modifications are reported to have opposing effects on gene expression [28]. However, both these modifications can simultaneously exist on the same histone tail [19]. We were therefore interested to investigate whether there was a correlation in the levels of both modifications across the *ANK1* locus. We found a positive correlation between average H3K4me3 and H3K27me3 levels across the six regions (r = 0.32, p = 0.002) (Supplementary Figure 3).

### H3K4me3 levels are correlated with DNA modification levels in the *ANK1* gene

**Figure 2.**
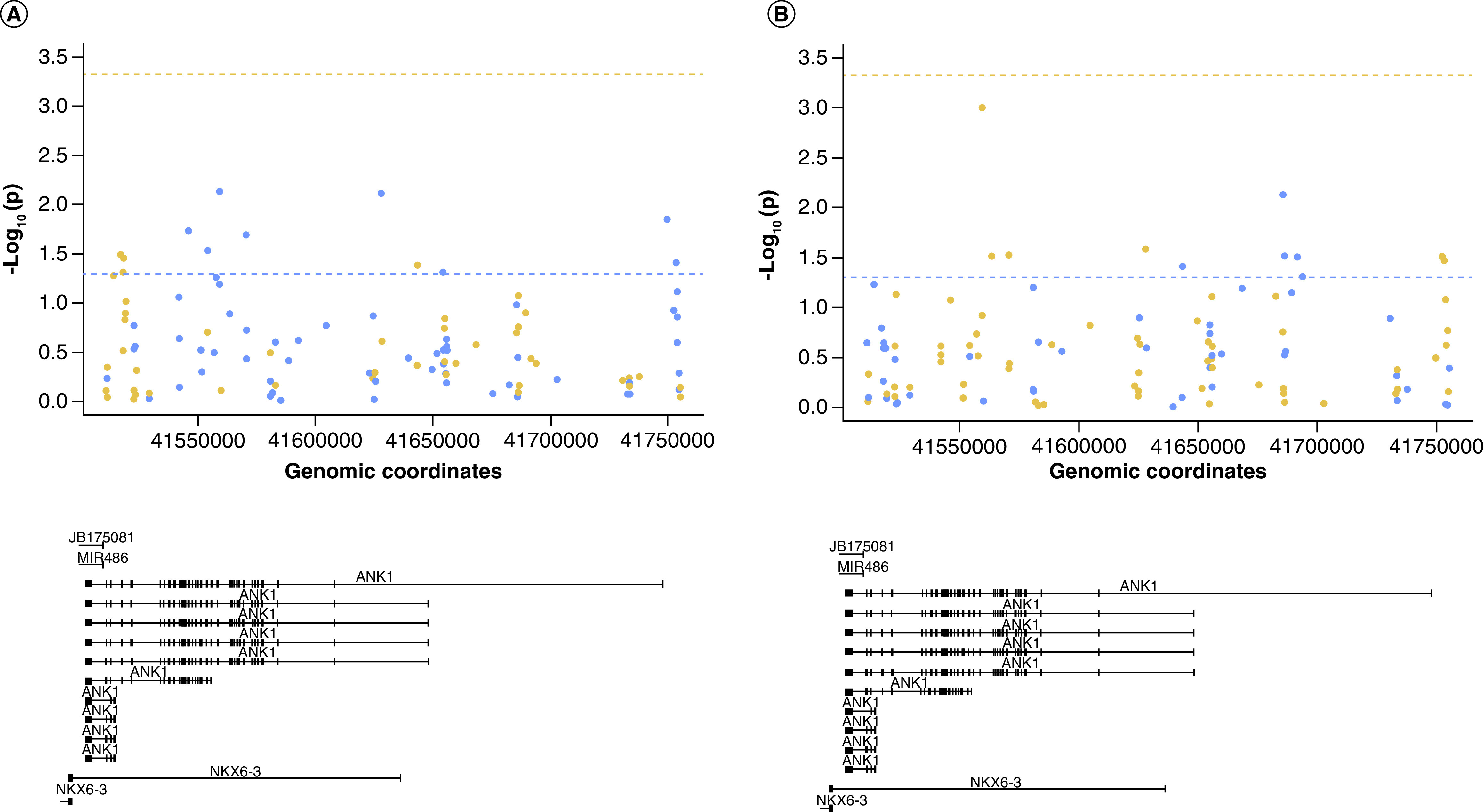
H3K4me3 levels are correlated with DNA modification levels in the *ANK1* gene. Shown is the correlation p-value (-log10(p)) between average H3K4me3 levels across the six regions tested and **(A)** DNA methylation levels or **(B)** DNA hydroxymethylation levels at specific CpG sites covered by the 450K array in relation to *ANK1* genomic location (bottom panel). The dashed horizontal line represents p = 0.05 (blue) and Bonferroni significance threshold (orange). Points are colored by correlation coefficient (r) >0 (blue) and r <0 (orange). No sites reached the Bonferroni significance threshold.

For a subset of samples we had already collected DNA methylation (5mC) and hydroxymethylation data (5hmC) [5]. Previous reports have demonstrated 5mC and H3K4me3 are negatively correlated with each other [29]. To further examine the relationship between histone modifications and DNA modifications, we first examined the correlation between average H3K4me3 levels across all six regions assayed and 5mC or 5hmC level at individual 450K derived CpG sites within the *ANK1* gene (n = 107 probes). This showed that DNA methylation at 11% of CpG sites (12/107) within the *ANK1* gene was nominally significantly correlated with H3K4me3 levels averaged across the six (primer Set) regions, although none reached Bonferroni significance (p < 4.67 × 10^-4^) (Figure 2A, Supplementary Table 4). Similarly, 5hmC at 10% of CpG sites (11/107) was nominally significantly correlated with H3K4me3 levels averaged across all six regions, with none reaching Bonferroni significance (Figure 2B, Supplementary Table 5). Five CpG sites were common to both analyses (cg08786207, cg08521995, cg13152952, cg09405790 and cg17256609), with an opposite direction of correlation seen for 5mC and 5hmC at each of these sites.

We then assessed the correlation between H3K4me3 levels in each individual region covered by the primers and 5mC or 5hmC levels at CpG sites covered by the 450K array in the same region (± 1 kb) to see whether localized correlations existed. One of the six regions showed a Bonferroni significant correlation (p < 8.3 × 10^-3^) between H3K4me3 levels and 5mC levels (chr8:41516703-41516777 (± 1 kb) (primer Set 1, Supplementary Figure 4A, r = -0.35, p = 0.005; chr8: 41519216-41519359 (± 1 kb), with a further two regions showing a nominally significant correlation (primer Set 2, Supplementary Figure 4B, r = -0.27, p = 0.039; chr8:41519342-41519460 (± 1 kb) (primer Set 3: Supplementary Figure 4C, r = -0.37, p = 0.033). No significant correlation was seen between H3K4me3 levels and 5hmC levels in the individual regions.

Finally, two specific DNA methylation sites in *ANK1*, cg11823178 (chr8:41519399) and cg05066959 (chr8:41519308), have been robustly shown to be hypermethylated with increasing AD neuropathology [5,7,8]. We were interested to investigate whether H3K4me3 levels were correlated with DNA modification levels at these two specific sites, given that primer Set 2 (chr8:41519216-41519359) covered cg05066959 and primer Set 3 (chr8:41519342-41519460) covered cg11823178. This showed a Bonferroni significant negative correlation of H3K4me3 levels across chr8:41519342-41519460 (primer Set 3) with DNA methylation levels at chr8:41519399 (cg11823178) (r = -0.36, p = 0.003). There was no correlation between H3K4me3 levels across that region and DNA hydroxymethylation levels. There was no significant correlation between H3K4me3 levels across chr8:41519342-41519460 (Primer Set 2) with 5mC or 5hmC levels at chr8:41519308 (cg05066959).

### H3K27me3 levels are correlated with DNA modification levels in the *ANK1* gene

**Figure 3.**
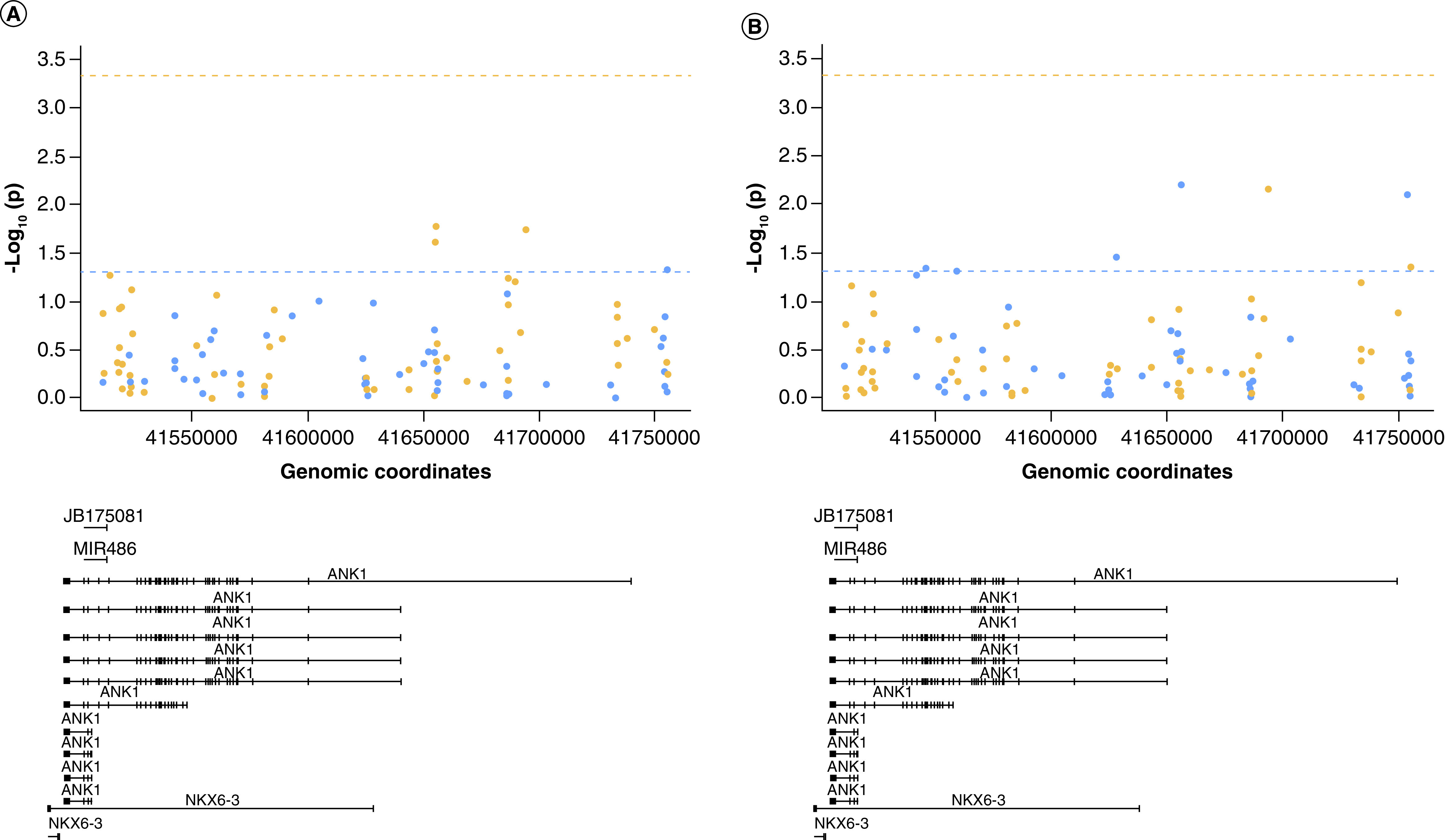
H3K27me3 levels are correlated with DNA modification levels in the *ANK1* gene. Shown is the correlation p-value (-log10(p)) between average H3K27me3 levels across the six regions tested and **(A)** DNA methylation levels or **(B)** DNA hydroxymethylation levels at specific CpG sites covered by the 450K array in relation to *ANK1* genomic location (bottom panel). The dashed horizontal line represents p = 0.05 (blue) and Bonferroni significance threshold (orange). Points are colored by correlation coefficient (r) >0 (blue) and r <0 (orange). No sites reached the Bonferroni significance threshold.

Next, we were interested to investigate whether a similar relationship with DNA modifications was observed for H3K27me3. First, we assessed the correlation between H3K27me3 levels averaged across all six regions assayed and the average DNA methylation or hydroxymethylation level at the 450K array-derived CpG sites on the *ANK1* gene (107 probes). This showed that DNA methylation levels at four of the CpG sites (Figure 3A & Supplementary Table 6) and DNA hydroxymethylation levels at seven of the CpG sites (Figure 3B & Supplementary Table 7) within the *ANK1* gene were nominally significantly correlated with average H3K27me3 levels, although none reached Bonferroni significance (p < 4.67 × 10^-4^). Only one probe was common to both analyses (cg22845790), with an opposite direction of correlation observed for 5mC (r = -0.30, p = 0.018) and 5hmC (r = 0.34, p = 0.007).

When we looked within the individual regions assayed, we observed no significant correlation between either 5mC or 5hmC levels with H3K27me3 levels in each individual region (± 1 kb). Similarly, 5mC and 5hmC levels at the two methylation sites in *ANK1* that are known to be hypermethylated in AD (chr8:41519308 – cg05066959 and chr8:41519399 – cg11823178) were not correlated with H3K27me3 levels across chr8:41519216-41519359 (primer Set 2) and chr8:41519342-41519460 (primer Set 3), respectively.

### H3K4me3 & H3K27me3 correlations with H3K27ac

Using matched data from Marzi *et al.* [23], we first examined whether there was a correlation of H3K27ac with H3K4me3 in any of the six regions we studied, and whether there was an interaction with the degree of pathology (Supplementary Table 8). We observed a Bonferroni significant correlation of H3K27ac with H3K4me3 at the genomic coordinates chr8:41519342-41519460 (primer Set 3: p = 3.38 × 10^-3^), with an interaction with pathology group, showing a stronger correlation in individuals with high pathology (p = 5.75 × 10^-3^). We observed no significant association of H3K27ac with H3K4me3 levels at any of the other five regions analyzed. Similarly, when we correlated H3K27ac with H3K27me3 we observed a Bonferroni significant correlation in two genomic regions: chr8:41519342-41519460 (primer Set 3: p = 3.31 × 10^-3^) and chr8:41625416-41625491 (primer set 4: p = 3.97 × 10^-3^), although there was only a nominally significant interaction with pathology in these two regions (primer Set 3: p = 0.03, primer Set 4: p = 0.01). There was no significant association of H3K27ac and H3K27me3 in any of the other four regions we quantified (Supplementary Table 9).

## Discussion

In this study, we used ChIP-qPCR to quantify two histone modifications, H3K4me3, (a marker of gene expression) and H3K27me3 (a marker of gene repression), across six regions of the *ANK1* gene in human, post-mortem brain samples.

We first investigated whether *ANK1* H3K4me3 and H3K27me3 levels are altered in the EC of individuals with a higher amount of AD neuropathology, compared with those with lower levels. When averaged across all genomic regions we quantified, we observed significantly less H3K4me3 (meeting our Bonferroni significance threshold), with no change in H3K27me3 in individuals with higher levels of neuropathology (Braak stage IV and above). A decrease in H3K4me3 would usually be associated with decreased gene expression and would suggest that *ANK1* gene expression would be reduced in individuals with AD neuropathology. However, a number of studies indicate that *ANK1* gene expression is increased in AD; Mastroeni and colleagues recently showed increased levels of *ANK1* gene expression in microglia in AD brain, although they showed no change in the astrocytes or neurons [30]. Whilst another study has shown that only certain transcript variants have increased expression in AD, with other variants having no disease-associated changes, although that study was undertaken on bulk tissue [7]. In our study, we have analysed H3K4me3 and H3K27me3 in bulk EC tissue. Different patterns of histone modifications are observed in different cell types, limiting our interpretation of the likely downstream consequence of these alterations on gene expression. In addition, as differing modification states will exist even within cells of the same type, as each will have been exposed to different stimuli, this could contribute a further small level of variation. In the future, studies of histone modifications and gene expression in parallel should be performed in specific cell populations sorted from post-mortem tissue to determine if the changes we have observed are ubiquitous across cell types or are specific to certain cell types.

We have shown H3K4me3 and H3K27me3 levels across the *ANK1* gene are positively correlated in post-mortem EC brain tissue. H3K4me3 and H3K27me3 are associated with active and inactive gene transcription, respectively [28]. It is likely that the positive correlation of H3K4me3 and H3K27me3 in our study in post-mortem brain samples results from a cell type-specific pattern of histone modifications as we have used bulk tissue containing multiple cell types. However, both these modifications have been shown to simultaneously exist on the same histone tail of genomes of ESCs [19] and in other cell types, including neuronal cells, at different stages of development [31,32] and so it is therefore possible that both these modifications are positively correlated within the same cell type.

There is evidence of cross-talk between DNA methylation and histone modifications [33]. For example, during embryo implantation, CpG island methylation is protected from removal, through a H3K4 methylation-dependent mechanism. In addition, 5mC acts as a template for histone modification patterns after DNA replication, mediated through methylcytosine-binding proteins that are capable of recruiting histone deacetylases [34,35] and methyltransferases [36]. In this study cytosine modification (5mC and 5hmC) levels at several specific loci were found to correlate with average histone modification (H3K4me3 and H3K27me3) levels across the *ANK1* gene, although none reached the Bonferroni significance threshold. H3K4me3 has been previously suggested to prevent DNA methylation at both CpG-island and non-CpG island start sites [29]. In our study, H3K4me3 levels were nominally significantly correlated with 5mC levels at 12 CpG sites, and 5hmC levels at 11 CpG sites, with five CpG sites common to both analyses and showing opposite directions of correlations for H3K4me3 with 5mC and 5hmC. In contrast to previous negative associations reported between H3K4me3 and 5mC [37], our study shows equal amounts of positive and negative association, dependent upon genomic location. Again, this could be due to different cell types being present in the samples between datasets, but could also represent 5mC site specific histone associations that have not yet been characterized. H3K27me3 levels were nominally significantly correlated with 5mC levels at four CpG sites and 5hmC levels at seven CpG sites, with one locus common to both analyses and showing an opposite direction of correlation for H3K27me3 with 5mC and 5hmC. Interestingly, 5hmC levels at four CpG sites (cg08786207, cg08521995, cg22845790 and cg090450790) were nominally significantly correlated with both H3K4me3 and H3K27me3 levels. Three of the probes showed an opposite direction of correlation with H3K4me3 to H3K27me3, as would be expected, given that they are markers of gene expression and repression, respectively. However, 5hmC levels at one locus (cg22845790) showed a positive correlation with both H3K4me3 and H3K27me3 levels. However, no correlation between 5mc or 5hmc and the histone modification level met our Bonferroni significant threshold, indicating that these findings could be false positives and require further studies with higher numbers of samples to replicate this. It was previously thought that 5hmC was a transient epigenetic mark and did not play a role in transcriptional regulation. 5hmC has been previously identified at high levels in the developing [38] and adult brain [39], particularly in neurons [40] and, as such, represents an important epigenetic mark to profile in the context of neurodegenerative diseases. One study has shown that 5hmC levels in ESCs are more strongly correlated with levels of other histone modifications (i.e., H3K4me1, H3K4me2, H3K18ac, H3K27ac, H4K5ac), than they are with H3K4me3 or H3K27me3 [41]. Our study shows only certain sites of methylation/hydroxymethylation are having an interaction with the H3K4me3 and H3K27me3 modifications at the *ANK1* locus. These key sites could be specific regulatory elements, however, to assess this further, studies designed to manipulate specific DNA methylation signals and then subsequently profiling histone modifications will be needed.

DNA methylation levels at two CpG sites, chr8:41519399 (cg11823178) and chr8:41519308 (cg05066959) have been previously reported to be hypermethylated in AD in numerous published studies [6–8]. Interestingly, we found H3K4me3 levels across chr8:41519342-41519460 (primer Set 3) to be significantly decreased in individuals with high neuropathology and to be negatively correlated with 5mC levels at chr8:41519399 (cg11823178). It has previously been suggested that H3K4 modifications are a preventative mechanism for *de novo* promoter cytosine methylation [42]. A proposed mechanism for this stems from the discovery that Dnmt3L, a Dnmt3 associated protein required for efficient *de novo* cytosine methylation, contains a domain that specifically interacts with unmodified H3K4, the binding of which is inhibited by methylation of the H3 tail [43]. Furthermore, mutation of this same domain in Dnmt3L leads to reduced 5mC levels [44]. These observations suggest that H3K4 methylation may play a role in blocking *de novo* DNA methylation at some genomic loci [43–45]. It is therefore a possibility that the decreased H3K4me3 levels in this region are facilitating the increased DNA methylation at chr8:41519399 (cg11823178) in AD. However, as we did not see any association of H3K4me3 levels across chr8:41519216-41519359 (primer Set 2) with 5mC at chr8:41519308 (cg05066959), it is difficult to draw any firm conclusions as to whether hypermethylation at chr8:41519399 in AD is caused by the reduction in H3K4me3 levels, or *vice versa*. In the future it will be of interest to quantify the levels of other histone modifications in the *ANK1* gene, as well as relating this to gene expression changes.

Looking to the future a more comprehensive study of more histone modifications in *ANK1* in AD should be undertaken, with these levels being integrated with levels of 5mC, 5hmC and miRNAs to build up a complete picture of the epigenetic landscape of the *ANK1* gene in AD. Ideally this would be performed in cell sorted, post-mortem brain tissue, however so far suitability of such tissue for ChIP studies has not been achieved. It is not possible to determine the order of events leading up to the changes in H3K4me3 in AD that have been reported here. It is feasible that changes reported here are a result of, rather than the cause of, AD pathology. In the future it will be of interest to study the temporal pattern and order of epigenetic changes in AD. Studies looking to epigenetically profile a large cohort of post-mortem brain samples with varying Braak staging for both cytosine and histone modifications could establish the order of epigenetic events in relation to pathology.

H3K4me3 has been previously shown (with H3K9me1) to be predictive of expression levels in low CpG content promoters (LCPs), while H3K27ac and H4K20me1 are predictive of high CpG content promoters (HCPs) [46]. To date, the only AD EWAS of histone modifications have quantified histone acetylation (H3K9ac and H3K27ac) [22,23], and no study has investigated genome-wide alterations in histone methylation patterns. In our study we have covered areas of known H3K4me3 and H3K27me3 peaks in the brain using qPCR assays. However, this method still only profiles a small amount of a relatively large (244 kb) gene. As such, in the future an H3K4me3 and H3K27me3 EWAS in AD using ChIP-Seq would be the optimal next step to completely characterize the levels of these histone modifications in AD in *ANK1*.

Our study provides a preliminary exploration of the histone modification profile of the *ANK1* gene in AD brain tissue. The differences in H3K4me3 levels identified in this paper, build upon the growing association of epigenetic alterations of the *ANK1* gene in AD. Although more studies are needed to replicate our findings and to determine whether epigenetic alterations in *ANK1* are causal in AD pathology, the consistent reporting of *ANK1* epigenetic alterations in AD to date, highlights it as an exciting area for further research. Functionally, we hypothesize that the *ANK1* epigenetic differences in disease are resulting in a change in *ANK1* gene expression. *ANK1* transcriptional alterations in the brain would likely impact the function of microglial cells, given the previously reported four-fold change in *ANK1* expression in AD microglia [30]. We hypothesize that, given *ANK1*‘s proposed function, this will result in dysregulation of the binding of the cytoskeleton to the plasma membrane of microglia.

## Conclusion

This study is the first to interrogate H3K4me3 and H3K27me3 modification profiles across the *ANK1* gene in AD brain tissue. Overall, these results suggest that H3K4me3 levels are reduced in multiple genomic regions in *ANK1* in AD EC and are correlated with changes in DNA methylation and hydroxymethylation. These patterns of epigenetic changes, together with increased DNA methylation, would be expected to result in reduced gene activation, which should be fully explored in future studies.

## Future perspective

Since the first EWAS of DNA methylation in AD, the amount of research exploring the role of epigenetic mechanisms in neurological diseases has increased dramatically. This has enabled the identification of robust disease-associated epigenetic changes in AD, including DNA methylation and histone modifications. Of these, *ANK1* is one of the most replicated differentially methylated genes identified in AD brain. This, coupled with the histone modification analysis we present here, further reinforces the importance of *ANK1* in AD etiology. However, there are still several research questions to be addressed, each with associated practical and analytical challenges. Epigenetic mechanisms by their nature are cell type specific. Future studies exploring these mechanisms will therefore need to be performed on isolated cell populations, rather than bulk tissue, establishing the cell type or types driving this AD association. Furthermore, functional characterization of the *ANK1* gene is still required to determine the role of this protein in the brain as well as the consequence of these epigenetic changes on phenotype. Answering these key questions will begin to address epigenetic causality of *ANK1* in AD.

**Summary points**

**Alzheimer's disease (AD)**

- AD is the most prevalent neurodegenerative disorder affecting ∼30 million people worldwide.
- It is characterized by accumulation of amyloid-β plaques and neurofibrillary Tau tangles within the brain.

**Epigenome-wide association studies (EWAS) and AD**

- A number of studies have highlighted robust differential DNA methylation differences at the *ANK1* locus in AD.
- Alterations in histone modifications have been associated with AD; two recent papers have found histone acetylation differences with disease.

***ANK1* has decreased levels of H3K4me3 in individuals with high neuropathology**

- Four of the six regions assayed showed a significant decrease in the level of H3K4me3 between individuals with low and high AD neuropathological burden, with two regions passing the Bonferroni significance threshold.
- Average H3K4me3 levels had a Bonferroni significant decrease in cases with high AD neuropathology compared with those with low neuropathology.

**H3K4me3 and H3K27me3 levels are correlated in post-mortem brain samples**

- H3K4me3 and H3K27me3 levels were positively correlated.

**H3K4me3 levels are correlated with DNA modification levels in the *ANK1* gene**

- DNA methylation at 11% of CpG sites within the *ANK1* gene was nominally significantly correlated with average H3K4me3 levels.
- DNA hydroxymethylation at 10% of CpG sites within the *ANK1* gene was nominally significantly correlated with average H3K4me3 levels.
- The previously identified differentially methylated site at Chr8:41519399 in *ANK1* showed a significant negative correlation with H3K4me3 levels across this region.

**H3K27me3 levels are correlated with DNA modification levels in the *ANK1* gene**

- DNA methylation levels at four of the CpG sites within the *ANK1* gene were nominally significantly correlated with average H3K27me3 levels.
- DNA hydroxymethylation levels at seven of the CpG sites within the *ANK1* gene were nominally significantly correlated with average H3K27me3 levels.

**H3K27ac is correlated with H3K27me3 & H3K4me3 levels**

- H3K27ac is correlated with H3K27me3 and H3K4me3 levels in the region covered by primer Set 3.

## References

[1] Prince M, Anders W, Maëlenn G, Gemma-Claire A, Yu-Tzu W, Matthew P. World Alzheimer Report 2015 - The Global Impact of Dementia, an analysis of prevalence, incidence, cost and trends. Alzheimer's Dis. Int. 1–87 (2015).

[2] Smith RG, Lunnon K. DNA modifications and Alzheimer's disease. Adv. Exp. Med. Biol. 978, 303–319 (2017). 28523553 10.1007/978-3-319-53889-1_16

[3] Lunnon K, Mill J. Epigenetic studies in Alzheimer's disease: current findings, caveats, and considerations for future studies. Am. J. Med. Genet. B Neuropsychiatr. Genet. 162B(8), 789–799 (2013). 24038819 10.1002/ajmg.b.32201 PMC3947441

[4] Roubroeks JaY, Smith RG, Van Den Hove DLA, Lunnon K. Epigenetics and DNA methylomic profiling in Alzheimer's disease and other neurodegenerative diseases. J. Neurochem. 143(2), 158–170 (2017). 28805248 10.1111/jnc.14148

[5] Smith AR, Smith RG, Pishva E Parallel profiling of DNA methylation and hydroxymethylation highlights neuropathology-associated epigenetic variation in Alzheimer's disease. Clin. Epigenetics 11(1), 52 (2019). 30898171 10.1186/s13148-019-0636-y PMC6429761

[6] Smith RG, Hannon E, De Jager PL Elevated DNA methylation across a 48-kb region spanning the HOXA gene cluster is associated with Alzheimer's disease neuropathology. Alzheimers Dement. 14(12), 1580–1588 (2018). 29550519 10.1016/j.jalz.2018.01.017 PMC6438205

[7] Lunnon K, Smith R, Hannon E Methylomic profiling implicates cortical deregulation of ANK1 in Alzheimer's disease. Nat. Neurosci. 17(9), 1164–1170 (2014). 25129077 10.1038/nn.3782 PMC4410018

[8] De Jager PL, Srivastava G, Lunnon K Alzheimer's disease: early alterations in brain DNA methylation at ANK1, BIN1, RHBDF2 and other loci. Nat. Neurosci. 17(9), 1156–1163 (2014). 25129075 10.1038/nn.3786 PMC4292795

[9] Lardenoije R, Roubroeks JaY, Pishva E Alzheimer's disease-associated (hydroxy)methylomic changes in the brain and blood. Clin. Epigenetics 11(1), 164 (2019). 31775875 10.1186/s13148-019-0755-5 PMC6880587

[10] Smith RG, Pishva E, Shireby G Meta-analysis of epigenome-wide association studies in Alzheimer's disease highlights novel differentially methylated loci across cortex. bioRxiv (2020). 10.1038/s41467-021-23243-4 PMC8192929 34112773

[11] Smith AR, Smith RG, Burrage J A cross-brain regions study of ANK1 DNA methylation in different neurodegenerative diseases. Neurobiol. Aging 74, 70–76 (2019). 30439595 10.1016/j.neurobiolaging.2018.09.024

[12] Cedar H, Bergman Y. Linking DNA methylation and histone modification: patterns and paradigms. Nat. Rev. Genet. 10(5), 295–304 (2009). 19308066 10.1038/nrg2540

[13] Sadakierska-Chudy A, Filip M. A comprehensive view of the epigenetic landscape. Part II: histone post-translational modification, nucleosome level, and chromatin regulation by ncRNAs. Neurotox Res. 27, 172–197 (2015). 25516120 10.1007/s12640-014-9508-6 PMC4300421

[14] Santos-Rosa H, Schneider R, Bannister AJ Active genes are tri-methylated at K4 of histone H3. Nature 419(6905), 407–411 (2002). 12353038 10.1038/nature01080

[15] Barski A, Cuddapah S, Cui K High-resolution profiling of histone methylations in the human genome. Cell 129(4), 823–837 (2007). 17512414 10.1016/j.cell.2007.05.009

[16] Boyer LA, Plath K, Zeitlinger J Polycomb complexes repress developmental regulators in murine embryonic stem cells. Nature 441(7091), 349–353 (2006). 16625203 10.1038/nature04733

[17] Lee TI, Jenner RG, Boyer LA Control of developmental regulators by polycomb in human embryonic stem cells. Cell 125(2), 301–313 (2006). 16630818 10.1016/j.cell.2006.02.043 PMC3773330

[18] Kouzarides T. Chromatin modifications and their function. Cell 128 (2007). 10.1016/j.cell.2007.02.005 17320507

[19] Bernstein BE, Kamal M, Lindblad-Toh K Genomic maps and comparative analysis of histone modifications in human and mouse. Cell 120(2), 169–181 (2005). 15680324 10.1016/j.cell.2005.01.001

[20] Kim HS, Kim EM, Kim NJ Inhibition of histone deacetylation enhances the neurotoxicity induced by the c-terminal fragments of amyloid precursor protein. J. Neurosci. Res. 75(1), 117–124 (2004). 14689454 10.1002/jnr.10845

[21] Guan J-S, Haggarty SJ, Giacometti E HDAC2 negatively regulates memory formation and synaptic plasticity. Nature 459, 55 (2009). 19424149 10.1038/nature07925 PMC3498958

[22] Klein HU, Mccabe C, Gjoneska E Epigenome-wide study uncovers large-scale changes in histone acetylation driven by tau pathology in aging and Alzheimer's human brains. Nat. Neurosci. 22(1), 37–46 (2019). 30559478 10.1038/s41593-018-0291-1 PMC6516529

[23] Marzi SJ, Leung SK, Ribarska T A histone acetylome-wide association study of Alzheimer's disease identifies disease-associated H3K27ac differences in the entorhinal cortex. Nat. Neurosci. 21(11), 1618–1627 (2018). 30349106 10.1038/s41593-018-0253-7

[24] Yocum AO, Steiner LA, Seidel NE A tissue-specific chromatin loop activates the erythroid ankyrin-1 promoter. Blood 120(17), 3586–3593 (2012). 22968456 10.1182/blood-2012-08-450262 PMC3482866

[25] Untergasser A, Cutcutache I, Koressaar T Primer3–new capabilities and interfaces. Nucleic Acids Res. 40(15), (2012). 10.1093/nar/gks596 PMC3424584 22730293

[26] Taberlay Phillippa C, Kelly Theresa K, Liu C-C Polycomb-repressed genes have permissive enhancers that initiate reprogramming. Cell 147(6), 1283–1294 (2011). 22153073 10.1016/j.cell.2011.10.040 PMC3240866

[27] Vienna RDCT. R Foundation for Statistical Computing (2012).

[28] Harikumar A, Meshorer E. Chromatin remodeling and bivalent histone modifications in embryonic stem cells. EMBO Rep. 16(12), 1609–1619 (2015). 26553936 10.15252/embr.201541011 PMC4693513

[29] Balasubramanian D, Akhtar-Zaidi B, Song L H3K4me3 inversely correlates with DNA methylation at a large class of non-CpG-island-containing start sites. Genome Med. 4(5), 47 (2012). 22640407 10.1186/gm346 PMC3506913

[30] Mastroeni D, Sekar S, Nolz J ANK1 is up-regulated in laser captured microglia in Alzheimer's brain; the importance of addressing cellular heterogeneity. PloS one 12(7), e0177814 (2017). 28700589 10.1371/journal.pone.0177814 PMC5507536

[31] Golebiewska A, Atkinson SP, Lako M, Armstrong L. Epigenetic landscaping during hESC differentiation to neural cells. Stem cells (Dayton, Ohio) 27(6), 1298–1308 (2009). 10.1002/stem.59 19489095

[32] Pan G, Tian S, Nie J Whole-genome analysis of histone H3 lysine 4 and lysine 27 methylation in human embryonic stem cells. Cell Stem Cell 1(3), 299–312 (2007). 18371364 10.1016/j.stem.2007.08.003

[33] Kondo Y. Epigenetic cross-talk between DNA methylation and histone modifications in human cancers. Yonsei Med. J. 50(4), 455–463 (2009). 19718392 10.3349/ymj.2009.50.4.455 PMC2730606

[34] Nan X, Ng HH, Johnson CA Transcriptional repression by the methyl-CpG-binding protein MeCP2 involves a histone deacetylase complex. Nature 393(6683), 386–389 (1998). 9620804 10.1038/30764

[35] Chi P, Allis CD, Wang GG. Covalent histone modifications–miswritten, misinterpreted and mis-erased in human cancers. Nat. Rev. Cancer 10(7), 457–469 (2010). 20574448 10.1038/nrc2876 PMC3262678

[36] Jin B, Li Y, Robertson KD. DNA methylation: superior or subordinate in the epigenetic hierarchy? Genes Cancer 2(6), 607–617 (2011). 21941617 10.1177/1947601910393957 PMC3174260

[37] Maunakea AK, Nagarajan RP, Bilenky M Conserved role of intragenic DNA methylation in regulating alternative promoters. Nature 466(7303), 253–257 (2010). 20613842 10.1038/nature09165 PMC3998662

[38] Spiers H, Hannon E, Schalkwyk LC, Bray NJ, Mill J. 5-hydroxymethylcytosine is highly dynamic across human fetal brain development. BMC Genomics 18(1), 738 (2017). 28923016 10.1186/s12864-017-4091-x PMC5604137

[39] Munzel M, Globisch D, Bruckl T Quantification of the sixth DNA base hydroxymethylcytosine in the brain. Angew Chem. Int. Ed. Engl. 49(31), 5375–5377 (2010). 20583021 10.1002/anie.201002033

[40] Kriaucionis S, Heintz N. The nuclear DNA base 5-hydroxymethylcytosine is present in Purkinje neurons and the brain. Science 324(5929), 929–930 (2009). 19372393 10.1126/science.1169786 PMC3263819

[41] Szulwach KE, Li X, Li Y Integrating 5-hydroxymethylcytosine into the epigenomic landscape of human embryonic stem cells. PLoS Genet. 7(6), e1002154 (2011). 21731508 10.1371/journal.pgen.1002154 PMC3121778

[42] Weber M, Hellmann I, Stadler MB Distribution, silencing potential and evolutionary impact of promoter DNA methylation in the human genome. Nat. Genet. 39(4), 457–466 (2007). 17334365 10.1038/ng1990

[43] Ooi SK, Qiu C, Bernstein E DNMT3L connects unmethylated lysine 4 of histone H3 to *de novo* methylation of DNA. Nature 448(7154), 714–717 (2007). 17687327 10.1038/nature05987 PMC2650820

[44] Rose NR, Klose RJ. Understanding the relationship between DNA methylation and histone lysine methylation. Biochim. Biophys. Acta 1839(12), 1362–1372 (2014). 24560929 10.1016/j.bbagrm.2014.02.007 PMC4316174

[45] Otani J, Nankumo T, Arita K, Inamoto S, Ariyoshi M, Shirakawa M. Structural basis for recognition of H3K4 methylation status by the DNA methyltransferase 3A ATRX-DNMT3-DNMT3L domain. EMBO Rep. 10(11), 1235–1241 (2009). 19834512 10.1038/embor.2009.218 PMC2775176

[46] Karlić R, Chung H-R, Lasserre J, Vlahoviček K, Vingron M. Histone modification levels are predictive for gene expression. Proc. Natl Acad. Sci. USA 107(7), 2926–2931 (2010). 20133639 10.1073/pnas.0909344107 PMC2814872

