# Supplementary material for: The histone modification H3K4me3 is altered at the ANK1 locus in Alzheimer's disease brain
Source: Future Sci OA. 2021 Feb 9;7(4):FSO665. doi: 10.2144/fsoa-2020-0161 (PMC8015672; doi:10.2144/fsoa-2020-0161)
Supplement: Supplementary file 1 [file fsoa-07-665-s1.docx]

**SUPPLEMENTARY FIGURES**


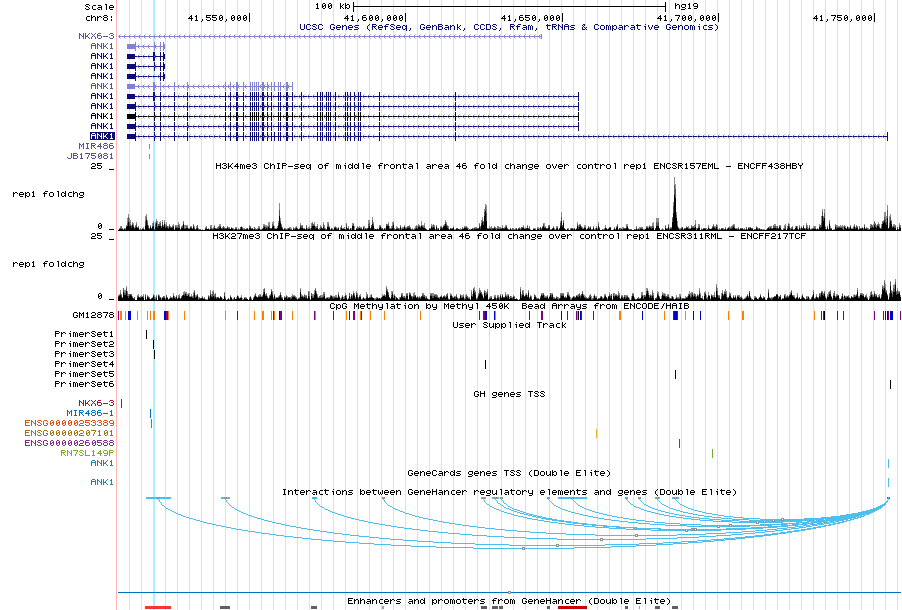


ENCODE H3K4me3 data

ENCODE H3K27me3 data

Illumina 450K CpG probes

qPCR primer locations

*ANK1* transcripts

**Supplementary Figure 1: Genomic regions in *ANK1* covered by ChIP-qPCR primer sets used in this study.** Displayed is the complete *ANK1* gene with all 10 transcript variants (shown in purple), established H3K4me3 and H3K27me3 profiles in ENCODE frontal cortex data (shown in black), locations of 450K methylation probes in the region, locations of all 6 primer sets (shown in black), and the location of previously identified DMR covered by the Illumina 450K methylation array including cg05066959 & cg11823178 (shown as a light blue line).

**
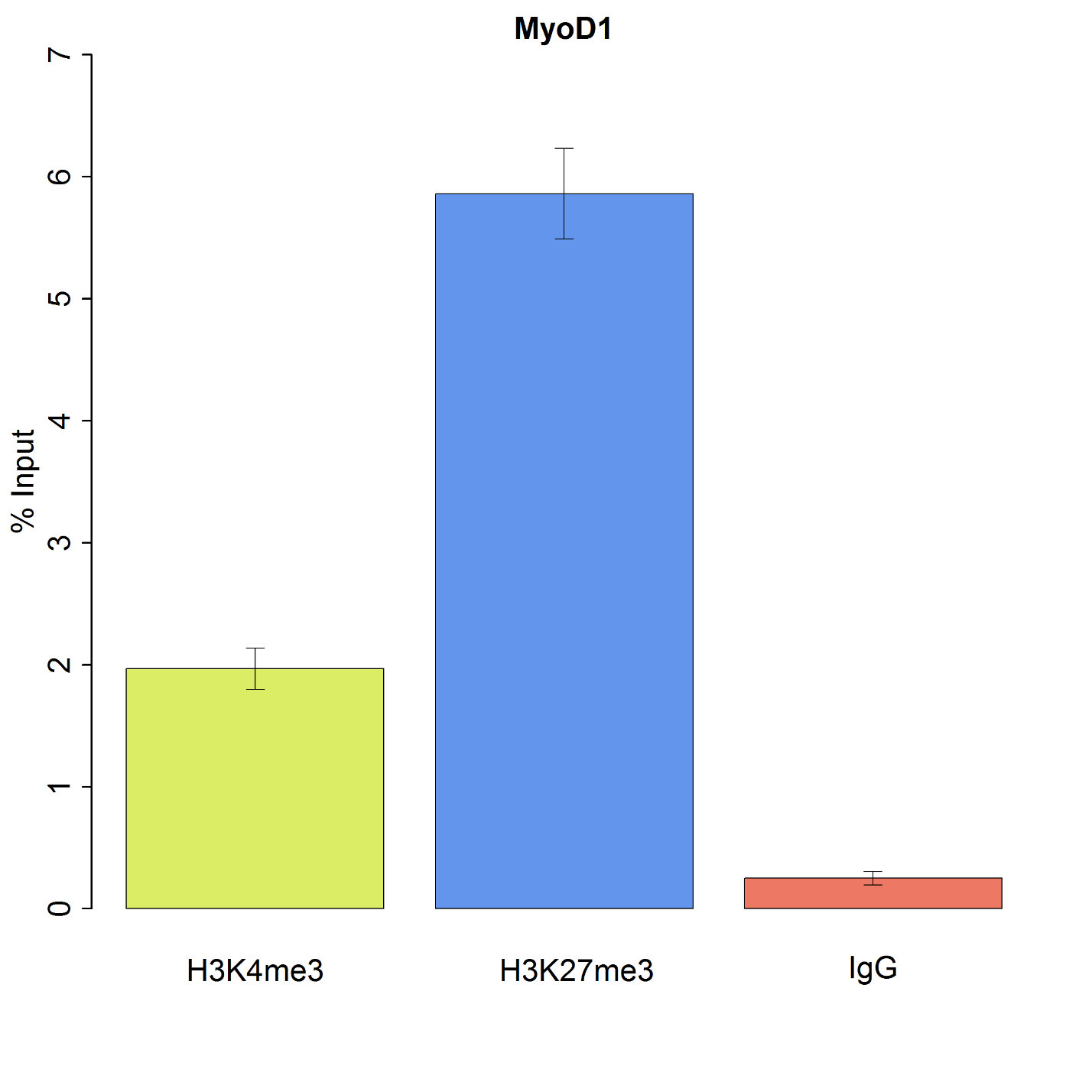
**

**Supplementary Figure 2: MyoD1 shows high H3K27me3 levels.**  MyoD1 was used as a positive control for H3K27me3 as previous studies have shown this to be a genomic region with high modification peaks. As expected we found high levels of H3K27me3 in this region. Data is represented as a percentage relative to input (% input).

**
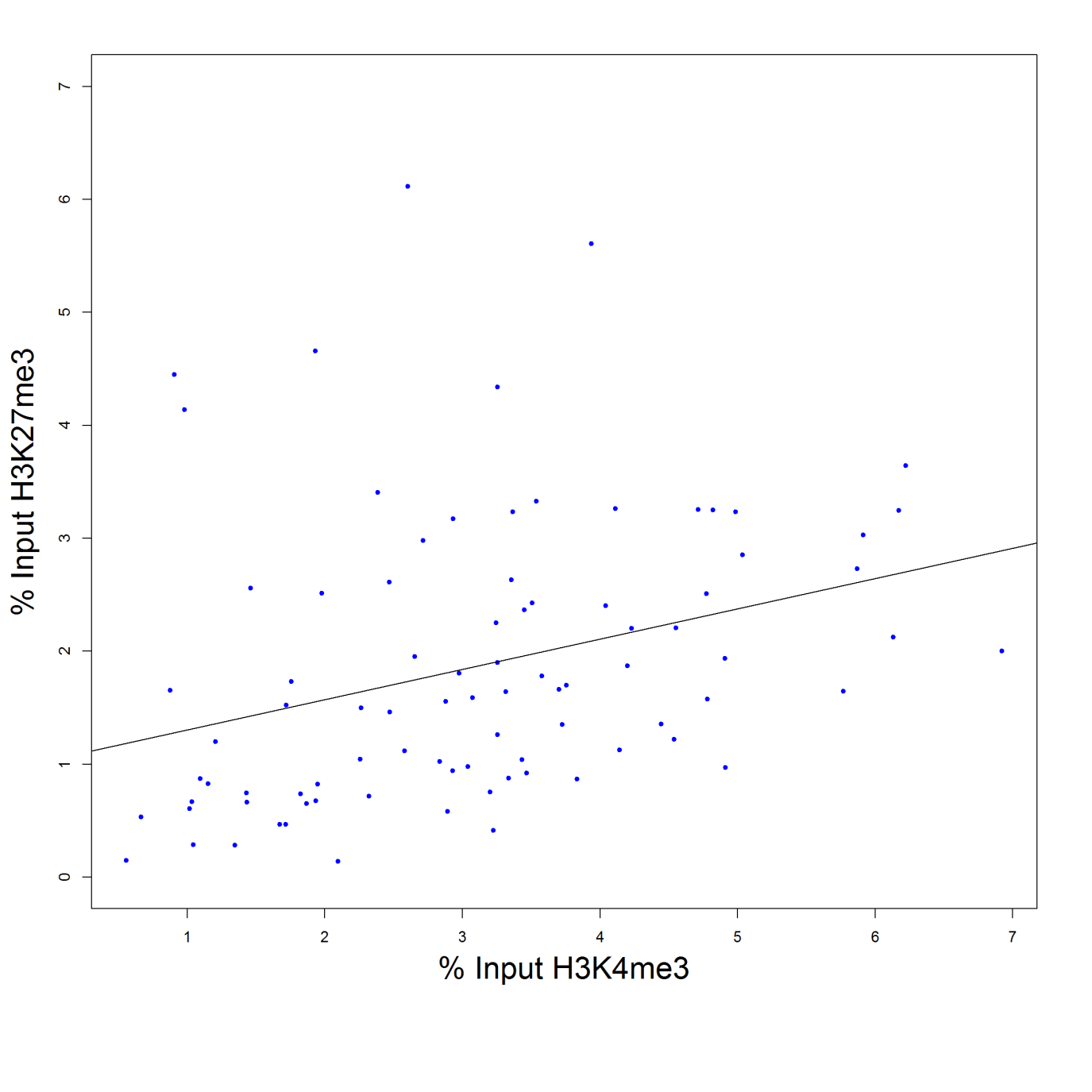
**

**Supplementary Figure 3: H3K4me3 levels are significantly correlated with H3K27me3 levels.** A Pearson’s correlation shows H3K4me3 and H3K27me3 (averaged across the six genomic regions) are significantly correlated with each other (*r* = 0.32, *P* = 0.002).


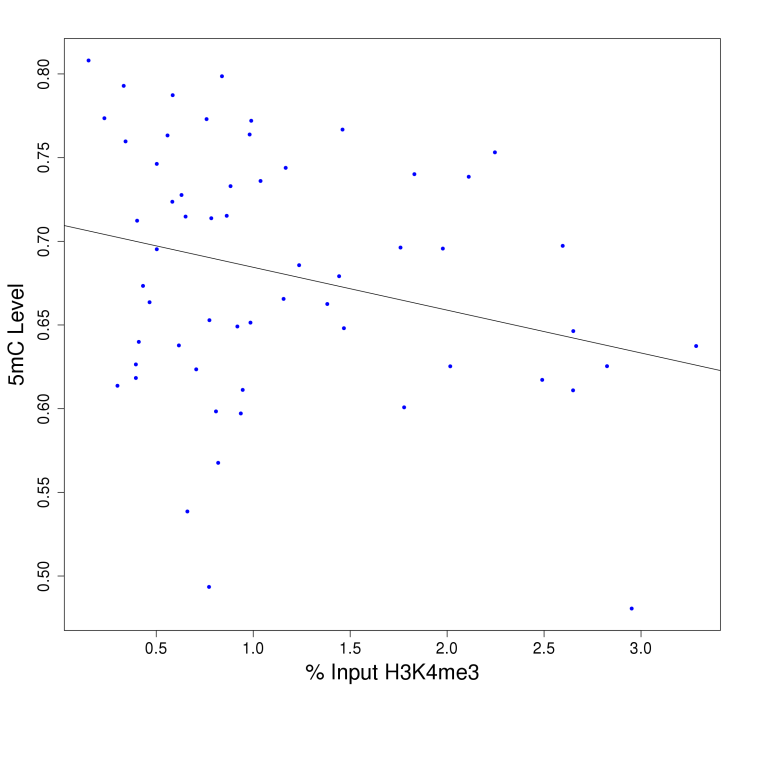

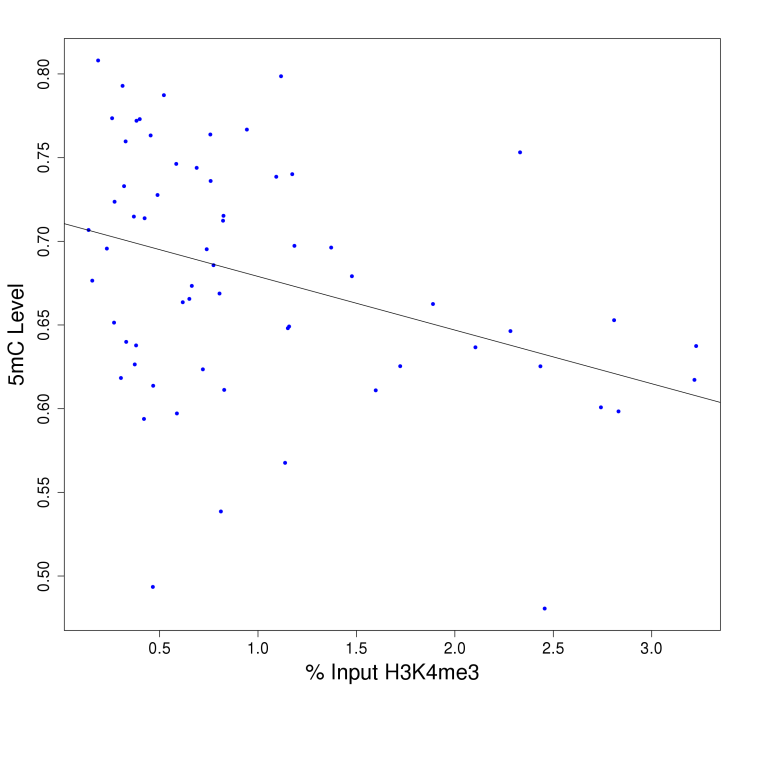


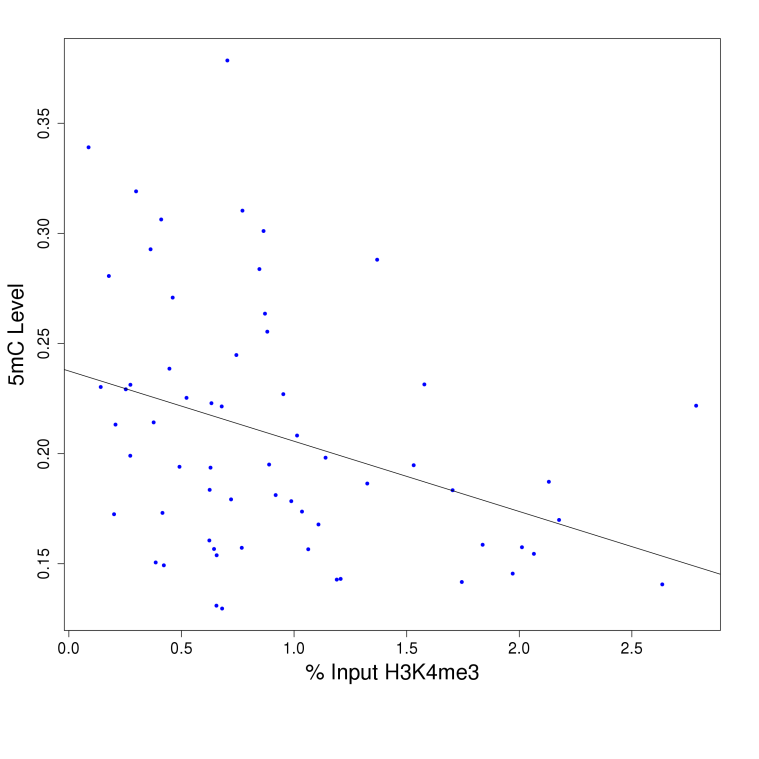


**A**

**B**

**C**

% methylation

% methylation

% methylation

**Supplementary Figure 4: H3K4me3 Levels are significantly correlated with DNA methylation levels** A Pearson’s correlation shows H3K4me3 levels at three of the six genomic regions interrogated were significantly correlated with 5mC levels in those regions (± 1kb), including a Bonferroni significant correlation at (**A**) chr8:41516703-41516777 (Primer Set 1): *r* = -0.35, *P* = 0.005, and a nominally significant correlation at (**B**) chr8: 41519216-41519359 (Primer Set 2): *r* = -0.27, *P* = 0.039, and (**C**) chr8:41519342-41519460 (Primer Set 3): *r*=-0.37, *P* = 0.033.
