# Supplementary material for: The histone modification H3K4me3 is altered at the ANK1 locus in Alzheimer's disease brain
Source: Future Sci OA. 2021 Feb 9;7(4):FSO665. doi: 10.2144/fsoa-2020-0161 (PMC8015672; doi:10.2144/fsoa-2020-0161)
Supplement: Supplementary file 2 [file fsoa-07-665-s2.docx]

**SUPPLEMENTARY TABLES**

|  | Low pathology | High pathology |
| --- | --- | --- |
| Braak stage | 0-III | IV-VI |
| Number of samples | 29 | 59 |
| Sex (M/F) | 14/15 | 35/24 |
| Mean age (±SD) | 79.2 (11.2) | 82.2 (8.2) |

**Supplementary Table 1: Demographic information for brain samples used in the study.** The samples were split in to two groups based on low pathology (Braak stage 0-III) and high pathology (Braak stage IV-VI). Shown for each group are the number of samples, number of males (M) and females (F) and mean age (± standard deviation (SD)).

| Primer Name | Genomic Coordinates | Position | qPCR Primers (5' to 3') | Tm (°C) | Product length (bp) | Efficiency | Final Concentration |
| --- | --- | --- | --- | --- | --- | --- | --- |
| ANK1 Primer Set 1 | chr8:41516703-41516777 | F | TAAGGAACATCCCAGCCTTC | 59.1 | 75 | 87% | 2 μM |
|  |  | R | TGTTTAGAATGGCCCCTGAG | 60.1 |  |  |  |
| ANK1 Primer Set 2 | chr8:41519216-41519359 | F | GCTCATAAAGAGGTGAGTGGAG | 58.1 | 144 | 89% | 2 μM |
|  |  | R | AGCTGGAGGTCGATATTGATTAC | 58.7 |  |  |  |
| ANK1 Primer Set 3 | chr8:41519342-41519460 | F | CAATATCGACCTCCAGCTCAC | 59.7 | 119 | 95% | 3 μM |
|  |  | R | GATCATTCGCAAGGTGGTTC | 60.5 |  |  |  |
| ANK1 Primer Set 4 | chr8:41625416-41625491 | F | CCGATTTGCATGTTTTGAGC | 61.5 | 76 | 93% | 2 μM |
|  |  | R | CTCAGGCAGAATCTGGAATAGG | 60.2 |  |  |  |
| ANK1 Primer Set 5 | chr8:41686181-41686328 | F | TGGTTCGCACTACAATGACC | 59.6 | 148 | 97% | 2 μM |
|  |  | R | TCGCGTGGAAAATCTTCAG | 59.9 |  |  |  |
| ANK1 Primer Set 6 | chr8:41754877-41755012 | F | GAGCTTGATTTGCCCCTTTC | 61.1 | 136 | 100% | 2 μM |
|  |  | R | CGGGGACCTACTTCCTTTTC | 59.9 |  |  |  |
| MyoD1 Primer Set 1 | chr11:17741321-17741428 | F | CGCCAGGATATGGAGCTACT | 59.3 | 108 | 95% | 2 μM |
|  |  | R | CGGGTCGTCATAGAAGTCGT | 60.1 |  |  |  |

**Supplementary Table 2:** **ChIP-qPCR primers used in this study.** Seven ChIP-qPCR primer sets were used in this study as they met our criteria for inclusion (single product, 85-115% efficiency). This included six qPCR primer sets specific to ANK1 and one set specific to MyoD1, which was used as a control set. The MyoD1 primer design was obtained from (23). Shown for each primer are genomic coordinates of the amplicon covered, melting temperature (Tm) for each primer, product length of amplicon in basepairs (bp), efficiency of primer, and final concentration of primers used.

| **Genomic region** | **Modification** | **FC** | **∆** | ***P*** |
| --- | --- | --- | --- | --- |
| chr8:41516703-41516777 (Primer set 1) | H3K4me3 | -0.08 | -0.09 | 0.541 |
|  | H3K27me3 | 0.36 | 0.36 | 0.138 |
|  | IgG | 1.54 | 0.15 | 0.228 |
| chr8:41519216-41519359 (Primer set 2) | H3K4me3 | -0.19 | -0.25 | 0.160 |
|  | H3K27me3 | 0.14 | 0.22 | 0.394 |
|  | IgG | 0.57 | 0.11 | 0.361 |
| chr8:41519342-41519460 (Primer set 3) | H3K4me3 | -0.30 | -0.41 | 0.033 |
|  | H3K27me3 | 0.07 | 0.11 | 0.770 |
|  | IgG | 0.03 | 0.01 | 0.947 |
| chr8:41625416-41625491 (Primer set 4) | H3K4me3 | **-0.31** | **-1.60** | **0.004** |
|  | H3K27me3 | 0.01 | 0.02 | 0.949 |
|  | IgG | 1.18 | 0.10 | 0.096 |
| chr8:41686181-41686328 (Primer set 5) | H3K4me3 | -0.22 | -2.53 | 0.023 |
|  | H3K27me3 | 0.29 | 0.49 | 0.178 |
|  | IgG | 1.00 | 0.33 | 0.243 |
| chr8:41754877-41755012 (Primer set 6) | H3K4me3 | **-0.39** | **-0.91** | **0.002** |
|  | H3K27me3 | 0.02 | 0.08 | 0.884 |
|  | IgG | 0.57 | 0.13 | 0.383 |

**Supplementary Table 3: H3K4me3 levels are decreased in specific regions of the *ANK1* gene in AD.** Shown for each genomic region assayed is the corrected difference (Δ) in histone modification levels relative to input and corresponding P value between individuals with low levels of pathology (Braak 0-III) and those with high levels of pathology (Braak IV-VI) after adjusting for the covariates of age, sex and batch. A positive fold change (FC) or Δ reflects increased levels of the modification with increasing pathology. When data is Bonferroni significant it is shown in bold.

| **Probe** | **Genomic Coordinates** | ***r*** | ***P*** |
| --- | --- | --- | --- |
| cg12148515 | chr8:41517301 | -0.27 | 0.032 |
| cg10794439 | chr8:41518051 | -0.25 | 0.048 |
| cg10715527 | chr8:41518139 | -0.27 | 0.035 |
| cg22532314 | chr8:41546072 | 0.30 | 0.019 |
| cg09576978 | chr8:41554080 | 0.27 | 0.029 |
| cg08786207 | chr8:41559303 | 0.33 | 0.007 |
| cg13152952 | chr8:41570554 | 0.29 | 0.021 |
| cg08521995 | chr8:41628237 | 0.33 | 0.008 |
| cg17256609 | chr8:41643375 | -0.26 | 0.041 |
| cg08923054 | chr8:41654455 | 0.25 | 0.048 |
| cg22746182 | chr8:41749867 | 0.31 | 0.014 |
| cg09405790 | chr8:41753440 | 0.26 | 0.038 |

**Supplementary Table 4: Correlation of H3K4me3 levels with DNA methylation levels at specific CpG sites.** Shown for each 450K array probe is genomic location (hg19), correlation coefficient (*r*) and corresponding *P* value for the correlation between average H3K4me3 levels across the six regions tested and DNA methylation levels at individual CpG sites. Data is only shown for the 12 CpG sites that showed a nominally significant correlation. No sites showed a Bonferroni significant correlation.

| **Probe** | **Genomic Coordinates** | ***r*** | ***P*** |
| --- | --- | --- | --- |
| cg08786207 | chr8:41559303 | -0.40 | 0.001 |
| cg07680965 | chr8:41563429 | -0.27 | 0.031 |
| cg13152952 | chr8:41570554 | -0.27 | 0.030 |
| cg08521995 | chr8:41628237 | -0.28 | 0.027 |
| cg17256609 | chr8:41643375 | 0.26 | 0.040 |
| cg19997384 | chr8:41685595 | 0.33 | 0.007 |
| cg16129172 | chr8:41686467 | 0.27 | 0.031 |
| cg14354820 | chr8:41691767 | 0.27 | 0.032 |
| cg22845790 | chr8:41694003 | 0.25 | 0.050 |
| cg05977053 | chr8:41752508 | -0.27 | 0.031 |
| cg09405790 | chr8:41753440 | -0.27 | 0.034 |

**Supplementary Table 5: Correlation of H3K4me3 levels with DNA hydroxymethylation levels at specific CpG sites.** Shown for each 450K array probe is genomic location (hg19), correlation coefficient (*r*) and corresponding *P* value for the correlation between average H3K4me3 levels across the six regions tested and DNA hydroxymethylation levels at individual CpG sites. Data is only shown for the 11 CpG sites that showed a nominally significant correlation. No sites showed a Bonferroni significant correlation.

| **Probe** | **Genomic Coordinates** | ***r*** | ***P*** |
| --- | --- | --- | --- |
| cg00176210 | chr8:41654967 | -0.28 | 0.025 |
| cg26326633 | chr8:41655078 | -0.30 | 0.017 |
| cg22845790 | chr8:41694003 | -0.30 | 0.018 |
| cg01616178 | chr8:41755140 | 0.25 | 0.047 |

**Supplementary Table 6: Correlation of H3K27me3 levels with DNA methylation levels at specific CpG sites.** Shown for each 450K array probe is genomic location (hg19), correlation coefficient (*r*) and corresponding *P* value for the correlation between average H3K27me3 levels across the six regions tested and DNA methylation levels at individual CpG sites. Data is only shown for the 4 CpG sites that that showed a nominally significant correlation. No sites showed a Bonferroni significant correlation.

| **Probe** | **Genomic Coordinates** | ***r*** | ***P*** |
| --- | --- | --- | --- |
| cg22532314 | chr8:41546072 | -0.25 | 0.047 |
| cg08786207 | chr8:41559303 | -0.25 | 0.049 |
| cg08521995 | chr8:41628237 | -0.27 | 0.036 |
| cg26984624 | chr8:41656079 | -0.34 | 0.006 |
| cg22845790 | chr8:41694003 | 0.34 | 0.007 |
| cg09405790 | chr8:41753440 | -0.33 | 0.008 |
| cg19844326 | chr8:41755409 | 0.25 | 0.045 |

**Supplementary Table 7: Correlation of H3K27me3 levels with DNA hydroxymethylation levels at specific CpG sites.** Shown for each 450K array probe is genomic location (hg19), correlation coefficient (*r*) and corresponding *P* value for the correlation between average H3K27me3 levels across the six regions tested and DNA hydroxymethylation levels at individual CpG sites. Data is only shown for the 4 CpG sites that showed a nominally significant correlation. No sites showed a Bonferroni significant correlation.

|  | **Correlation of H3K27ac vs H3K4me3** | | | | **Interaction of H3K27ac and pathology group vs H3K4me3** | |  |  |  |  |  |
| --- | --- | --- | --- | --- | --- | --- | --- | --- | --- | --- | --- |
| **Genomic region** | **Effect** | | **P** | | **Effect Interaction** | **P** |  |  |  |  |  |
| chr8:41516703-41516777 (Primer set 1) | -0.79 | | 0.75 | | 0.14 | 0.97 |  |  |  |  |  |
| chr8:41519216-41519359 (Primer set 2) | 1.67 | | 0.74 | | 3.35 | 0.53 |  |  |  |  |  |
| chr8:41519342-41519460 (Primer set 3) | 14.66 | | **3.38E-03** | | 18.69 | **5.75E-03** |  |  |  |  |  |
| chr8:41625416-41625491 (Primer set 4) | -3.06 | | 0.20 | | -3.80 | 0.16 |  |  |  |  |  |
| chr8:41686181-41686328 (Primer set 5) | 0.44 | | 0.85 | | -0.12 | 0.97 |  |  |  |  |  |
| chr8:41754877-41755012 (Primer set 6) | -5.78 | | 0.74 | | -13.70 | 0.48 |  |  |  |  |  |
|  |  |  | |  | | | |  |  |  |  |

**Supplementary Table 8: Correlations for H3K27ac levels with H3K4me3 in *ANK1*.** Shown for each genomic region is the effect and corresponding *P* value for the correlation between H3K27ac (measured by ChIP-seq) and each H3K4me3 (measured by ChIP-PCR) and the effect and corresponding *P* value for the interaction of H3K27me3 and pathology group with H3K4me3. Bonferroni significant correlations are shown in bold.

|  | **Correlation of H3K27ac vs H3K27me3** | | | | **Interaction of H3K27ac and pathology group vs H3K27me3** | |  |  |  |  |  |
| --- | --- | --- | --- | --- | --- | --- | --- | --- | --- | --- | --- |
| **Genomic region** | **Effect** | | **P** | | **Effect Interaction** | **P** |  |  |  |  |  |
| chr8:41516703-41516777 (Primer set 1) | -5.11 | | 0.06 | | -4.41 | 0.28 |  |  |  |  |  |
| chr8:41519216-41519359 (Primer set 2) | 4.55 | | 0.39 | | 6.70 | 0.24 |  |  |  |  |  |
| chr8:41519342-41519460 (Primer set 3) | 21.31 | | **3.31E-03** | | 20.61 | 0.03 |  |  |  |  |  |
| chr8:41625416-41625491 (Primer set 4) | -9.88 | | **3.97E-03** | | -10.35 | 0.01 |  |  |  |  |  |
| chr8:41686181-41686328 (Primer set 5) | -0.63 | | 0.67 | | -0.73 | 0.71 |  |  |  |  |  |
| chr8:41754877-41755012 (Primer set 6) | 4.39 | | 0.58 | | 6.15 | 0.48 |  |  |  |  |  |
|  |  |  | |  | | | |  |  |  |  |

**Supplementary Table 9: Correlations for H3K27ac levels with H3K27me3 in *ANK1*.** Shown for each genomic region is the effect and corresponding *P* value for the correlation between H3K27ac (measured by ChIP-seq) and each H3K27me3 (measured by ChIP-PCR) and the effect and corresponding *P* value for the interaction of H3K27me3 and pathology group with H3K27me3. Bonferroni significant correlations are shown in bold.
